# A Sub-Specialty for Women

**Published:** 1902-04

**Authors:** 


### Editorial.

### A SUB-SPECIALTY FOR WOMEN.

A valued contributor, Dr. Wright, of Cincinnati, has given his views in another part of this number on a new specialty for women connected with the practice of dentistry. This proposition is worthy of serious consideration, not alone from the fact that it emanates from an honored member of the profession, whose large experience entitles him to a patient hearing, but also that it belongs to a class of suggestions that mean an enlargement of the field of endeavor to a portion of the human family greatly in need of independent financial extension in their sphere of work. It is strictly upon the lines of the highest altruism, and the natural feeling is to unite with it and use all possible effort to hasten the day of its adoption in college work.

Many years have passed since the writer of this, in 1866, delivered an address before a dental graduating class and a large audience in Philadelphia, and then and there took for a part of his theme the admission of women into dentistry through the then closed doors of dental colleges. The suggestion at that period was so at variance with accepted thought and practice that the remarks and arguments adduced to sustain them simply roused the feeling of amusement in the minds of the auditors and indignation among the writer's colleagues; and when, subsequently (1869), he offered a resolution before the American Dental Association, "That in view of the successful results obtained in the education of women as dentists," they should be admitted to full membership in subordinate associations,—up to that time two dental colleges had admitted and graduated each one woman, —the resolution was, of course, at once tabled. Nothing else was expected or desired. These two apparently inappropriate efforts were made simply to rouse thought upon a vital subject, not then considered to any extent in or outside of the dental profession. Any radical suggestion that tends to change the current of accepted thought must slowly penetrate the barriers of prejudice. It is something gained to secure a hearing. While the resolution was unanimously laid on the table, it accomplished all the writer intended, and very shortly thereafter the doors of nearly all dental colleges were open to women, and they are welcome members to all societies, and are, at present, in the National Dental Association not only as members, but fill some important offices. It is probable that many now active would like to forget the narrow position they occupied thirty-six years ago. He would indeed be a bold man who would now question the right of any woman to graduation in dentistry or medicine.

Allusion is here made to this historical matter in order to show, by illustration, that the suggestion of Dr. Wright, however new and contrary to accepted thought it may be, may contain a germ of practical value that will be simply a forerunner of a great advance in prophylactic dentistry. It is certainly true that we are on the eve of a radical change in dental practice. Not that old methods will be given up, but that new ones will be added, not alone for the direct preservation of the teeth from caries, but in the treatment of the oral cavity to prevent contamination, through this source, of the entire physical organism. We, as dentists, have simply touched the outer borders of prophylaxis, and it has required the courageous work of a number of earnest men to rouse us to a conception of our duty in the premises. That the oral cavity is the main culture basin for pathogenic bacteria is well known, but how little has that fact been impressed on the minds of the present generation, either through dental or medical instruction. We must come to appreciate the fact that the mouth may be the open door for disease to enter the entire organism. When this is fully appreciated, the plan suggested by Dr. Wright may be adopted. So fully is the writer impressed with the importance of prophylactic treatment that he hesitates to offer any serious objections to the plan proposed, but that there are serious objections must be apparent. The plan means partial culture, and this is always a dangerous ground to occupy. The essayist suggests that colleges should "offer an opportunity for the partial and separate training," and that upon completion of one year of study and practice should be given a certificate of competence to practise. Admitting this to be practicable, we are left in a maze of uncertainty as to the final result of such partial education. Knowledge of human nature leads to the opinion that the majority receiving this certificate would soon become dissatisfied with the monotony of this operative procedure and gradually drift into general practice, thus involving us in a mixed profession and a lower standard. If all the legal difficulties could be overcome, such a sub-specialty would continue to be a standing menace to legitimate practice.

A comparison between these special students and the trained nurse in medicine cannot be truthfully drawn. The nurse could not by any possibility overstep the bounds of her profession and enter the practice of medicine upon her diploma, but it is imagined that the trained mouth specialist would be tempted to step over the border-line, and that without much regard to State laws or boards of examiners.

The writer is fully in accord with the idea that women are the proper agents for this modern prophylactic measure. To be effective it must begin early in life, and men are not well adapted by nature to work on children. The question involved is not so much one of sex, but is one of training. This should not be partial, but be made of equal thoroughness with that given to men; in fact, let women become dentists in the highest and best sense, and then make themselves specialists in this important work.

It must be clear that if the intelligent portion of the community had this matter thoroughly explained to them, there would be a rapid appreciation of its importance and an extensive demand for the services of carefully instructed women, but it is to be doubted whether these same people would be willing to permit their children's teeth to be cared for by one they know to be partially instructed. With the higher attainments outlined it might be possible to secure a house-to-house clientele, the only feasible method of reducing this part of operative dentistry to a system. The dentist in full practice would never be able to include this properly in his daily work.

The placing of the teeth and adjacent parts in a perfectly clean and healthy condition requires more real skill and practical knowledge than the placing in of a filling. This statement may be regarded as an exaggeration, but it is thought a careful consideration of all that this operation requires will convince the thoughtful mind that it is not an over-statement. Filling teeth is a purely mechanical operation, and, the foundation principles once acquired, it can be carried on continuously upon the same lines by one of ordinary mechanical ability. To clean teeth and place the adjacent structures in a normal healthy condition means not only an expert ability in the use of instruments, but a quite thorough knowledge of special pathology and therapeutics, and this can only be acquired by long months and, it may be, years of training.

While we may differ as to the methods of reaching a common and desired end, there can be no difference in regard to the importance of the main idea that this branch is particularly adapted for women, but let the ability be acquired by present methods. To accomplish this all dental colleges should be open to women. In the mean time the knowledge of the importance of oral sanitary conditions should be spread far and wide, that the new prophylaxis may become part of the training of every household and be as familiar to the child as the daily bath is in every well-regulated family.

